# Validation of a violence risk screening for youth in psychiatric inpatient care—a pilot study of V-RISK-Y

**DOI:** 10.3389/fpsyt.2023.1210871

**Published:** 2023-07-26

**Authors:** John Olav Roaldset, Carina C. Gustavsen, Øyvind Lockertsen, Torbjørn Landheim, Stål K. Bjørkly

**Affiliations:** ^1^Department of Mental Health and Addiction, Oslo University Hospital, Oslo, Norway; ^2^Department of Child and Adolescent Psychiatry, Oslo University Hospital, Oslo, Norway; ^3^Department of Nursing and Health Promotion, Oslo Metropolitan University, Oslo, Norway; ^4^Molde University College, Molde, Norway

**Keywords:** aggression, risk screener, violence threats, adolescent, acute psychiatry, self-perception, user participation

## Abstract

The reason for this study was the void of validated risk assessment screening tools for violence in adolescence psychiatry. Our aims were to test the predictive validity and feasibility of a pilot version of the Violence Risk Screening for Youth (V-RISK-Y). The V-RISK-Y was based on a violence risk screen for adults, the V-RISK-10, and adapted to adolescents, resulting in 12 risk items that are scored for (a) presence and (b) relevance for future violence. In this naturalistic, prospective observational study, the V-RISK-Y was scored at admission and compared with recorded episodes of violent acts and threats during hospital stay. The target population was all 92 patients admitted to the emergency department of adolescent psychiatry at Oslo University Hospital for 1 year, of which 67 patients were scored with the V-RISK-Y at admission and constituted the study sample. The predictive validity of the V-RISK-Y for violent behavior showed an AUC of 0.762 (*p* = 0.006). Staff approved the screener and found it to be equally or better usable than the V-RISK-10, which was previously used in the department. Still, a high proportion of raters failed to follow the scoring instructions of relevance scores, reducing feasibility. The results must be interpreted within the limits of a pilot study and low power. We conclude that results suggest changes of certain parts of the V-RISK-Y and provide a basis for testing a revised edition of the screener in a more comprehensive study, preferably with a multicenter design.

## 1. Introduction

Short checklists have been shown to produce positive results and thereby reduce risk in many areas, as, for example, in aviation, in complicated construction projects, and also in somatic medicine where a simple 90-s preoperative checklist reduced deaths and complications by over a third in eight major hospitals from five continents (1, 2). Three randomized controlled trials from Switzerland, the Netherlands, and Denmark showed that the use of coercive measures and violence was significantly reduced (30–70%) after an 8–24 h inpatient behavioral warning-signs checklist was implemented (3–5).

Instruments to assess risk for violence have been developed since the mid-1980s, mostly in adult forensic and prison psychiatry. They are comprehensive and facilitate recommendations for all types of further risk management including the use of restrictive means. Still, they are time-consuming and require special expertise (6, 7).

The need for shorter and simpler instruments to accommodate busy clinical inpatient settings led to the development of instruments for measuring patients’ potential for imminent violence (8–24 h), such as the Brøset Violence Checklist (BVC) and the Dynamic Appraisal of Situational Aggression (DASA) (8, 9), which are developed to monitor behavioral changes frequently observed to occur in the hours and days preceding a violent incident (e.g., confusion, irritability, and physical and verbal threats), and have content that appears more “causal”—that is, observable behaviors that are antecedent, proximate, and explanatory for subsequent violence. These types of tools are designed to accomplish three main clinical tasks: (1) predict aggression; (2) communicate risks across a treatment team, and (3) to distinguish who does vs. does not need to have a more comprehensive risk assessment completed.

Another brief screening checklist developed for general psychiatry was the V-RISK-10, a simple screener based on risk factors with a time horizon from days to a few months (10–12). A screening tool for violence is used to identify persons with a possible risk of violence, and the main purpose is to help identify who needs immediate measures to prevent violence and to distinguish who needs a more comprehensive risk assessment (13).

There are comprehensive and time-consuming instruments for risk assessment developed for child and adolescent psychiatry, such as the SAVRY (14), ERASOR (15), or START:AV (16). There has been research concerning short-term risk assessment instruments of children and youth in emergency departments (17, 18). Recently an English abstract of the Risk Screener Youth has been published in the Netherlands (19), but to the best of our knowledge, no other screener or short-term instruments for assessing the risk of violence in youths have been validated for clinical use (20). Given this lack, some emergency psychiatric departments for adolescents in and outside of Norway have used the V-RISK-10.

Violence risk screeners might be relevant for youths, for example in contexts where a youth is unknown, if there is a need for an assessment in a short time, if the time is inconvenient (evenings and nights, weekends, and holidays), or if access to professionals or comprehensive instruments is limited. The center for research and education in forensic psychiatry serving the South-eastern Norway Regional Health Authority has, on several occasions, been contacted with requests regarding violence risk screening for youth or inquiries about methods or instruments available for youths. Further, there has been requests from institutions in Sweden and the United States to the V-RISK-10 team in Norway for permission to change certain items of the V-RISK-10 to better suit young people.

This led to the development of the Violence Risk Screening for Youth aged 12–18 (V-RISK-Y), which lasted for a 3-year period from 2018 and was carried out in collaboration with departments and specialists in child and adolescent psychiatry in Norway, Sweden, Finland, and the United States. Because the V-RISK-10 was currently used in some adolescent departments, we took the 10 items of the V-RISK-10 as a starting point for the V-RISK-Y. Each of the 10 items was reviewed regarding whether it was relevant for youths or whether it should be changed and adapted to youths and their situations. Whether new items should be added was also considered. The V-RISK-Y is not a complete risk assessment or risk management tool, but a risk screener to help identify risk in different contexts at an early stage. It belongs to the structured professional judgment (SPJ) tradition of risk instruments, which is characterized by a two-step assessment combining the scoring of a structured risk instrument with a final individual risk estimate of the patient/client (7, 21).

Aims of this pilot study were to test (a) the predictive validity and (b) the feasibility of the V-RISK-Y in an inpatient emergency psychiatric unit for adolescents.

## 2. Methods

### 2.1. Design, setting, and participants

In this naturalistic prospective observational study, the V-RISK-Y was scored at admission and compared with recorded episodes of violence and threats during the hospital stay.

The study was conducted at the Emergency Unit, Department of Child and Adolescent Psychiatry at Oslo University Hospital in Norway (UPA), with a catchment area covering the city of Oslo with about 700,000 inhabitants. The target population was all young people 12–18 years of age who were admitted to the ward within a year (*n* = 92), from the 6th of May, 2020 and to the 5th May, 2021. Sixty-seven patients (73%) had sufficient data and constituted the study sample.

UPA has five beds and two shielding rooms (see 2.3.2.2.). They receive patients mainly from the municipal psychiatric emergency room, from youth psychiatric outpatient teams, and from the children’s welfare agency’s emergency service linked to Oslo University Hospital.

These young people present with a variety of clinical diagnoses and problems. Common to all is that they cannot be safely managed in the community and require inpatient admission.

The most common problems are mental disorders or conduct disorders combined with risk of suicide or violence.

Although we were not permitted to access the youth’s admitting diagnoses for the purposes of this project (in 2021), we know that the most common ICD-10 main diagnoses in the department in 2022 were F2 Psychoses followed by F5 Eating disorders, F8 Autism spectrum disorders, F3 Affective disorders, and F2 Anxiety disorders (8). Half of the patients had two or more ICD-10 diagnoses.

Half of the patients were admitted with self-harm or suicidal risk, 40% for investigation and treatment of illness or behavior, and 10% for acute problems related to eating disorders, such as forced feeding, severe malnutrition, or re-feeding syndrome.

### 2.2. Procedure

Before the study started, clinical staff were informed about the study and the V-RISK-Y.

Because the V-RISK-Y was to be tested under realistic circumstances, there was no special supervision or follow-up during the study; the V-RISK-Y is self-instructing and with all the necessary information on the form. Approximately, 75% of the staff had used the V-RISK-10 regularly as a compulsory part of the admission procedure during a 3-year period before the V-RISK-Y project started.

During the study period, the V-RISK-Y was scored by the staff after they had completed the clinical admission interview and without the presence of patient or parents. An interdisciplinary collaboration on the scoring was desirable (physician/psychologist and ward staff). The scores should be based on the present information from the referring authority, the clinical interview at admission, comparators, hospital records, and current observations. Efforts should not be forced to collect additional information. Patients who were not scored with the V-RISK-Y on admission were excluded from the study. Reasons for a missing score could be due to many simultaneous admissions, parallel acute situations in the department, or admissions in shifts with uncertainties about who is doing the V-RISK-Y. At admission or soon after, both patients and parents, if possible, got written and verbal information about the study.

All episodes of physical violence or threats of violence were recorded by the ward staff on a separate form, the Violence and Threats recording form (VT form; see 2.3.2).

During the project, staff members scored eight short case-stories to calculate the inter-rater reliability of the V-RISK-Y. At the end of the project, staff members filled in the “Evaluation form for staff” to give their feedback on the V-RISK-Y. All data were anonymized and transferred electronically to the research server at the Oslo University Hospital.

### 2.3. Measures

#### 2.3.1. V-RISK-Y (baseline variable)

The V-RISK-Y was developed with particular emphasis on emergency settings characterized by high patient turnover, time pressure, and patients being received around the clock, year around.

The forerunner, the V-RISK-10, was developed for general psychiatry with an emphasis on emergency contexts (10–12). There were three requirements for the tool: (1) Easy and short time to use around the clock all year round, (2) no pre-training was required, the form should be self-instructing, and (3) could be scored by inexperienced staff. A recent literature review found that the V-RISK-10 was among the most accurate instruments for predicting the risk of violence in an acute psychiatric context for adults (13).

All 10 items from the V-RISK-10 and any new items were thoroughly discussed with clinicians and researchers in Norway and abroad before decisions were made. Five of the items from the V-RISK-10 were retained unchanged in the V-RISK-Y: (1) Violent acts, previous or current, (2) Violent threats, previous or current, (3) Substance abuse, previous or current, (7) Suspicion, and (8) Lack of empathy (i.e., the youth expresses and/or exhibits behavior that shows a lack of insight to the thoughts and feelings of others). Three items were expanded to include the parents’ perceptions in addition to the youths’: (6) Lack of insight, (9) Unrealistic plans, and (10) Stress vulnerability. Two items were changed due to clinical and diagnostic differences between adults and youths (22): (4) Major mental illness, previous or current, was changed to (4) Severe mental symptoms, previous or current (i.e., the youth has displayed odd or inappropriate behavior and/or expressed thoughts that do not correspond with their developmental age. This includes expressed symptoms of anxiety, depression, autism spectrum disorders, and symptoms/disorders involving distorted perceptions of reality or similar violence). Lastly, (5) Personality disorders was changed to (5) Disruptive, impulsive behavior/Behavioral disorders. Research has shown an association between childhood adversities and later violent behavior during adolescence (39). Based on this, a new item was added, (11) Childhood adversities, previous or current. User (patient) participation has increasingly been emphasized and recommended in psychiatry, but this has been little emphasized for violence risk assessments. We found three articles concerning a patient’s own perception of violence risk, and all showed a positive correlation between user perception and later violence (23–25). In line with this, (12) Adolescent’s/parent’s own assessment of risk was added as the last item in V-RISK-Y.

An English version of the V-RISK-Y form can be downloaded for free from www.sifer.no.

#### 2.3.2. Presence scores on V-RISK-Y

All 12 items should be scored with respect to their presence with four scoring options: (1) No = do not fit, (2) Maybe/Moderate = fits maybe, or is present in moderately severe degree, (3) Yes = is present, and (4) Do not know = too little information, or conflicting information. The emergency unit receives many unknown patients, the accompanying information may be scarce, and the “admission interview” may also provide less information than required.

##### 2.3.2.1. Relevance scores on V-RISK-Y

**Figure 1:**
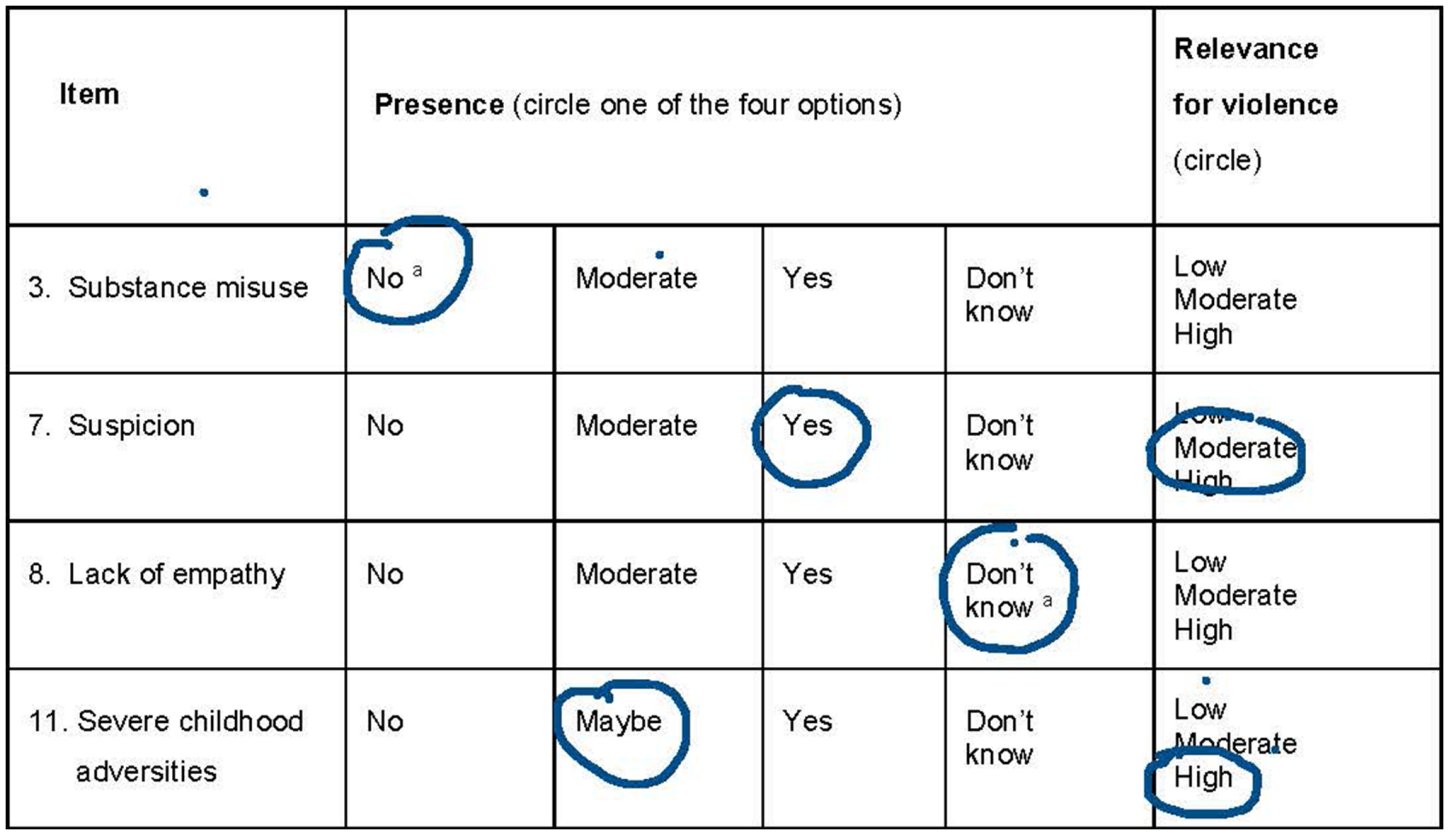
Example of scoring four of the items in V-RISK-Y. ^a^Presence scores of No (item 3) and Don’t know (Item 8) should not be scored for relevance.

In addition to its presence, each item should also be assessed for its relevance for future violence with three scoring options: (1) Low relevance, (2) Moderate relevance, and (3) High relevance. Relevance should only be assessed for items with presence scores of Maybe/Moderate or Yes (not for items with presence scores of No or Do not know). Figure 1 shows an example of scoring four of the items in the V-RISK-Y. Reasons for including the relevance for future violence were inspired by the HCR-20 version 3 (7). The use of that are considered relevant AND important when plans are to be made for restrictions, monitoring, and treatment. Even if it is less likely that there is sufficient information in the screening context to assess relevance with confidence, we nevertheless wanted to explore this possibility further in the pilot project.

##### 2.3.2.2. Overall risk assessment of low, moderate, or high scores

At the end of the V-RISK-Y form, a final risk assessment for each individual should be performed with three options: Low, Moderate, or High risk. The decision is based on the scores on the screens and all other available information. A decision was also to be taken on the need for further risk assessment and the implementation of immediate restrictive means, such as, for example, permanent watch and shielding. Shielding is defined as the confinement of patients to a single room or a separate unit/area inside the ward, accompanied by a member of staff. The rational for this is to improve treatment in a better context.

#### 2.3.3. Definition of violence and the violence and threats recording form (outcome variable)

Definition: Violence was defined as physical attacks (including with a harmful object or by arson) against another person to inflict physical injury or bodily harm (anger or damage of things was not included). Severe violent acts were defined to result in physical injury, sexual assault, and any assaultive act that involved the use of weapon. Moderate violent acts were defined to be kicks, blows, knocks, and pushes that did not cause physical injury. Violence also includes verbal and physical threats of violence against other people. Verbal threats were limited to threats to inflict physical harm to other people and included such threats via social media. Physical threats can be movements and gestures that signal physical attack. The definitions accord with international research (26).

The VT form was constructed for this project and was based on violence recording checklists from corresponding studies (27, 28). The VT form records verbal threats (including social media), physical threats, moderate violent acts, and severe violent acts during the hospital stay. Episodes in the hospital that take place before the V-RISK-Y is scored are not recorded in the VT form. The form contains detailed scoring instructions for each violence category. In addition, the location of the violent episode, time, and some characteristics of the victim(s) were recorded. Prior to the study, staff were trained in using the VT form. To avoid under-registration, the VT form was made very simple compared to existing instruments, such as the SOAS-R or REFA (29, 30). This accords with a previous study that found less under-recording of episodes when short and simple tools were used (31).

#### 2.3.4. Inter-rater reliability test

During the project, the psychologists, physicians, and ward staff who participated in the scoring of the V-RISK-Y were invited to score eight short de-identified case stories on an individual basis. The eight cases were constructed and had no connection to the patients in the project. The cases were available in the staff room throughout the project (only for ward staff), so you could score when there was an opportunity. The scoring was done on an individual basis and not in an interdisciplinary or team setting.

#### 2.3.5. Evaluation form

Toward the project’s end ward staff filled out a short, simple Evaluation form designed to map the time needed to fill out the V-RISK-Y and staff satisfaction with it, to compare the experiences with the V-RISK-Y with the prior use of V-RISK-10 and to provide an opportunity for feedback on experiences or suggestions for changes.

### 2.4. Data analyses

STATA 17 was used for intra class correlation analyses (ICC) and fractional polynomials. SPSS 26 was used for all other analyses. Mann–Whitney U-test, *t*-test, Fisher’s exact test, and chi-square test were used to compare groups. The area under the receiver operating characteristic (ROC) curve, or AUC, was used to determine overall predictive accuracy. AUC values range from 0 to 1, a value of 0.5 indicating by chance prediction, and a value of 1.0 indicating perfect prediction. The following predictive values were estimated for the cut-off giving the highest sum of sensitivity + specificity, when assuming sensitivity >80%; (a) sensitivity, (b) specificity, (c) positive predictive value (PPV), and (d) negative predictive value (NPV).

Binary logistic regression was used to compute the odds ratios for the summed total score, as well as the single item scores on the V-RISK-Y for violent episodes controlled for age and sex. With respect to single items analyses, we used No—Do not know—Moderate/Maybe—Yes (0-1-2-3) as a continuous explanatory variable. The linearity of this variable was tested by using fractional polynomials (32).

Internal consistency was analyzed by Cronbach’s alpha, and each single item’s contribution was analyzed by removing each one from the scale. The intra class correlation (ICC) was estimated to explore the inter-rater reliability (IRR) by using a linear mixed model (LMM). The design is unbalanced, since there are unequal numbers of observations for the raters.

Linear mixed model (LMM) is used since the scale is approximately linear.

To increase statistical power the four categories of violent threats and physical violence in the VT schemes were merged into one category, violence. One person counted only once in the statistics. For persons recorded with violence, the first hospital stay with violence was chosen as the index stay. For persons without violence, the first stay was chosen.

#### 2.4.1. Missing scores and do not know items

Missing scores on presence items were replaced using mode imputation to increase sample size.

This means that missing scores for an item were replaced with the most frequent value for that specific item. Only eight out of a total of 804 items were missing a score (12 items × 67 patients). Items 3, 7, 11, and 12 were missing on six forms. Of these, two forms had two missing and four forms had one missing item score. The No score was the most frequent score on these four items, so all imputed values were set to 0.

With respect to the relevance scores, only 16 forms were completed in accordance with the instructions. Forty-two of the 145 Moderate/Maybe presence scores (29%), and 55 of the 179 Yes scores (30%) lacked the corresponding relevance scores. According to the relevance instructions, the presence scores of No and Do not know should not be followed by scores for relevance for violence, however, 54 of the total 397 No scores (12%) and 44 of the total 118 Do not know scores (37%) were scored with Low, Moderate, or High relevance for violence. In 21 forms, relevance was scored for all present options (also No and Do not know scores), 15 forms had some relevance scores and some missing scores, and 15 forms had no relevance scores at all.

With respect to the single items in this study, 112 items (14%) were scored with Do not know, 128 (16%) with Moderate/Maybe, 174 (22%) with Yes, and 391 (49%) with No.

The handling of Do not know scores has been sparcely discussed in the literature. Former scoring manuals of the HCR-20 have ignored the Do not know scores when computing a sum score, based on: No = 0, Maybe/Moderate = 1, and Yes = 2 (33). A recent paper based on reanalysis of the data from validation studies of the V-RISK-10 in three acute psychiatric departments for adults (*n* = 1,500) found that Do not know scores represent a risk factor comparable with the Moderate/Maybe score and should not be ignored (34). Whether the same is the case for a youth psychiatric acute population, we cannot know for sure. Still, we have assumed that it is more likely that Do not know constitutes a risk factor, and that it cannot be ignored for this group either. Thus, the presence scores of all risk items were categorized in the following way in the analyses for the V-RISK-Y: No = 0, Do not know = 1, Maybe/Moderate = 2, and Yes = 3.

### 2.5. Ethical approval

The studies involving human participants were reviewed and approved by the data protection officer at Oslo University Hospital. The present study was approved without the patients’ or parents’/next to kins’ consent by the data protection officer at Oslo University Hospital (ID 20/01146). Because the study is low risk and did not involve direct patient interviewing, the research team sought permission to conduct the study without obtaining written informed consent from the youth or their caregivers. Further, concerns about the potential generalizability of the project contributed to this decision. For example, there might be some youths or parents who will not consent to participation, and, in some cases, informed consent could be difficult to obtain. Some people who have symptoms such as pronounced ambivalence, obsessive–compulsive disorder, or loss of reality may find it difficult to give consent because of the influence of their symptoms and functioning. They may spend a lot of time on the issue of consent, be insecure and suspicious about what consent will mean for them or be unable to form an opinion about the implications of consent. Some may be admitted against their will and become negative regarding the ward and unwilling to consent based on this. How many would not consent is difficult to predict, but the possibility that non-consenters have certain common characteristics (skewed selection, not random) will reduce generalizability.

At admission or soon after, both patients and parents got written and verbal information about the study. The patients or parents had no further contact with the study—no interviews and no forms to fill in.

## 3. Results

### 3.1. Demographic data

The study sample constituted 55 girls (82%) and 12 boys (18%). Mean age was 15.5 for girls and 16.1 for boys (*t* = −1.5, *p* = 0.219). Mean hospital stay was 14.7 days (range 1–71), for girls 14 days (range 1–60), and for boys 17 days (range 1–71; Mann–Whitney U *=* 253, *p* = 0.722). The mean and median sum-score of the V-RISK-Y were 13.2 and 12.0, respectively, *s.d.* = 7.2, and range was 2–29.

Violent acts and violent threats (violence) were recorded for 11 patients (16%), 7 girls (13%) and 4 boys (33%) (Fisher exact test, *p* = 0.099). The mean V-RISK-Y sum-score for youths recorded with violence was 19.6 compared with 12.0 for youths without violence, *t* = -2.7, *p* = 0.019. Two youths were recorded with severe violence (one boy), three with moderate violence (one boy), and six with threats (two boys). One boy was recorded with more than six violent episodes, one girl with three episodes, one boy and three girls with two episodes, and two boys and four girls with one violent episode, respectively.

Youths recorded with violence had significantly longer hospital stays than those without violence, 26 days versus 12 days (Mann-Whitney U = 300, *p* = 0.023), but mean age was not significant across youths with and without recorded violence, 14.8 years versus 15.6 years (*t* = -1.8, *p* = 0.073). In multivariate regression analysis, age, sex and hospital stay were all significant for violence, with Odds Ratios (95%) of 0.43 (0.22-0.84), *p* =0.014, 11 (1.3-92), *p* = 0,029 (boys), and 1.051 (1.003-1.101), p = 0.036, respectively.

Twenty-five (27%) of the 92 patients who were admitted were not scored with the V-RISK-Y at admission and were excluded. Mean age for the excluded subjects was 16.4 years versus 15.4 years for the study sample (*t* = 2.3, *p* = 0.023), mean length of hospital stay was 10.5 days (range 1-38) versus 14.7 days (1-71) (Mann-Whitney U = 550, *p* = 0.187, and 9 (43%) versus 12 (18%) were boys (Chi2 = 3,6, df = 1, *p* = 0.060), respectively.

### 3.2. Internal consistency

The internal consistency (Cronbach’s alpha) for the summed score of all the 12 V-RISK-Y items was 0.78 (95% CI 0.69–0.85, *p* < 0.001). When one item of the scale was deleted and the other entered, the highest value, alpha = 0.80, was obtained for Item 7 (Suspicion) and Item 11 (Severe trauma). The lowest value, alpha = 0.74, was obtained for Item 2 (Threats) and 12 (Youths or parents’ risk).

### 3.3. Inter-rater reliability

Thirty-three out of approximately 70 staff members (49%) independently scored the V-RISK-Y on eight short de-identified case stories. Each rater scored between one and six forms, and a total of 90 forms (34%) were completed. Only ward staff scored the cases. Eight missing scores were replaced by mode-imputation values. The mean V-RISK-Y summed total score of the eight case stories ranged between 18.4 and 25.0. Three forms concluded with Low risk, 53 with Moderate risk, and 30 with High risk.

One rater scored one case, eight raters scored two cases, two raters scored three, four, and five cases, respectively, and six raters scored six cases. Case 1 was scored by 17 raters, case 2 by 15, case 3 by 16, case 4 by 13, case 5 by nine, case 6 by nine, case 7 by 10, and case 8 by 10 raters, respectively.

The ICC for the summed total score was 0.51 and for the Low-Moderate/Maybe-High risk estimate, was 0.42. The ICC for single items were, respectively, 0.82 (Violent acts), 0.46 (Violent threats), 0.85 (Substance abuse), 0.66 (Severe psychiatric symptoms), 0.57 (Conduct/impulsive disorders), 0.30 (Lack of insight), 0.76 (Suspicion), 0.76 (Lack of empathy), 0.27 (Unrealistic plans), 0.76 (Stress exposure), 0.79 (Severe trauma), and 0.35 (User perception). The effect size of ICC < 0.50 is considered poor, 0.51–0.75 moderate, 0.76–0.90 good, and 0.91–1.0 excellent (35). A more liberal interpretation considers <0.40 as poor, 0.40–0.59 as fair, 0.60–0.74 as good, and 0.75–1.0 as excellent (36).

### 3.4. Predictive results

Area of the curve (AUC) values for presence scores of the V-RISK-Y are displayed in Table 1. The AUC for the V-RISK-Y was recalculated when Do not know = 0, showing a decrease from 0.762 (95% CI = 0.57–0.96), *p* = 0.006 to 0.741 (95% CI = 0.54–0.94), *p* = 0.012. Other predictive values were sensitivity 0.82, specificity 0.75, PPV 0.39, and NPV 0.95.

**Table 1:** Area under the curve of the receiver operating characteristics (AUC) for V-RISK-Y for violent behavior during hospitalization.

	AUC	(95% CI)	*p*
V-RISK-Y (all 12 items)	0.762	(0.566–0.958)	0.006
VY 10 items^a^	0.787	(0.601–0.973)	0.003
VP 11 items (VY 10 items^a^ + Item 11 childhood trauma)	0.759	(0.565–0.953)	0.006
VY 11 items (VY 10 items^a^ + Item 12 Adolescent’s/parent’s own risk assessment)	0.783	(0.595–0.970)	0.003
V-RISK-Y overall risk assessment after scoring low, moderate, or high risk for violence	0.779	(0.632–0.927)	0.004

^a^The 10 items from V-RISK-10 which were the basis for V-RISK-Y, of which five items were unchanged, and five items changed in the final V-RISK-Y.

Table 2 shows results from uni-and multivariate analyses (controlled for age and sex) of the summed total score of the V-RISK-Y (range 0–36), and the single items (range 0–3) as continuous variables. When hospital stay was added to age and sex as control factors, no single item was significant, except item 2 Violent threats (*p* = 0.038). The single items are ordinal variables (No—Do not know—Moderate/Maybe—Yes) but testing with fractional polynomials (32) showed that the ordinal variable fitted as a continuous variable (0-1-2-3) in this sample.

**Table 2:** Uni- and multivariate logistic regression analyses of the predictive validity of the sum-scores and of single items of V-RISK-Y for inpatient violent episodes adjusted for age and sex univariate analyses multivariate analyses adjusted for age and sex.

	Univariate analyses				Multivariate analyses adjusted for age and sex	
	OR^a^	95% CI	*p*	OR^a^	95% CI	*p*
Age^b^	0.596	0.363–0.978	**0.040**			
Sex^b^ (girls are reference)	5.40	1.06–27.4	**0.042**			
V-RISK-Y						
V-RISK-Y sum-score (12 items)^c^	1.16	1.05–1.28	**0.004**	1.14	1.02–1.29	**0.026**
VY-10 sum-score (10 items)^c,d^	1.21	1.07–1.37	**0.003**	1.21	1.04–1.40	**0.015**
Analyzes of the single items of V-RISK-Y^e^						
1. Violence	2.61	1.41–4.86	**0.002**	2.28	1.12–4.61	**0.023**
2. Threats	2.29	1.31–4.00	**0.004**	1.89	1.04–3.45	**0.038**
3. Substance abuse	0.951	0.403–2.25	0.951	1.08	0.398–2.90	0.887
4. Severe psychiatric symptoms	1.10	0.596–2.04	0.759	0.827	0.398–1.72	0.611
5. Disruptive, impulsive behavior/behavioral disorders	2.24	1.24–4.04	**0.008**	1.86	0.944–3.65	0.073
6. Lack of insight	1.57	0.874–2.81	0.131	1.53	0.795–2.95	0.203
7. Suspicion	2.15	1.25–3.68	**0.006**	2.43	1.24–4.73	**0.009**
8. Lack of empathy	1.84	0.978–3.45	0.059	1.65	0.800–3.39	0.176
9. Unrealistic plans	1.69	0.962–2.97	0.068	1.43	0.776–2.60	0.252
10. Stress vulnerability	1.23	0.653–2.31	0.524	1.24	0.590–2.62	0.567
11. Severe childhood adversities	0.619	0.328–1.17	0.139	0.626	0.312–1.26	0.188
12. Youth’s/parents’ own perception	2.18	1.27–3.76	**0.005**	1.79	0.993–3.22	0.053

^a^Odds ratio. ^b^Bivariate analysis. ^c^OR for a higher score on the scales with range 0–36 (VRISK-Y) and range 0–30 (VY-10), i.e., 0–1, 1–2, 2–3, etc. ^d^VY-10 is the 10 items from V-RISK-10 of which five were changed and five unchanged. ^e^OR for a higher score on the scale range 0–3, i.e., 0–1, 1–2, and 2–3. Bold values means that *p* < or = 0.050.

### 3.5. Other results

Table 3 presents a comparison of the overall V-RISK-Y risk estimate (Low, Moderate, High) with respect to violent episodes, decisions of restrictive means, and V-RISK-Y summed total scores.

**Table 3:** Comparison of overall risk assessment with respect to not violent patients, violent patients, V-RISK-Y sum-scores, and restrictive means.^a^

	Not violent patients	Violent patients	V-RISK-Y	(95% CI)	*p*	Restrictive means
	*n* = 56	*n* = 11	sum-score			*n* = 11
Overall risk assessment						
Low risk	42	2	9.6	(8.3–11)		0^b^
Moderate risk	10	8	20	(17–22)	< 0.001^c^	8 (44%) ^b^
High risk	3	1	27	(23–31)	<0.001^c^, 0.027^d^	3 (75%) ^b^

^a^Some variables have one or more missing scores, for example, one score is missing on the Low-Moderate-High risk scores for the Not violent patients group; ^b^Numbers (%) of patients imposed restrictive means in the Low, Moderate, and High groups, respectively; ^c^Compared with Low risk; and ^d^Compared with Moderate risk.

### 3.6. Staff evaluation of feasibility

Six physicians/psychologists and 10 ward staff filled in a short evaluation form. The anticipated mean time to score the V-RISK-Y was 6–7 min. Staff commented that it was difficult and time-consuming to rate relevance for violence, especially for unknown patients, and that the concept of relevance required more in-depth thinking and was difficult to assess in the admission situation. One staff rated the feasibility of the V-RISK-Y as moderate, 11 rated it as good, and 4 rated feasibility as very good. Six staff assessed the V-RISK-Y to be as useful as the V-RISK-10, the standard risk screener in the ward. Ten rated the V-RISK-Y as more useful. All 16 staff recommended the V-RISK-Y for use in emergency adolescent psychiatry.

## 4. Discussion

According to Rice and Harris (37), the AUC estimates obtained in this study would be considered large in magnitude, comparable to a Cohen’s *d* of 1.0. However, reliability is a pre-requisite of validity, and the poor to fair inter-rater reliability observed in our study weakens the significance of the results. Wide confidence intervals of the AUCs and the constraints of the pilot project design also places limitations on the strength of the results.

With these limitations in mind, we observed that the V-RISK-Y was characterized by high sensitivity which indicates good screening properties. The V-RISK-Y had high accuracy in identifying patients with no risk of violence (NPV), but lower accuracy in identifying patients at risk (PPV), in line with earlier results in research on risk screeners (12, 28, 38). The internal consistency of the screener was satisfactory.

Current results provide a basis for proceeding with a revised version of V-RISK-Y in a larger study.

In this study, the AUC for recorded violence was higher when Do not know = 1 compared to when Do not know = 0, which confirms Eriksen’s findings that Do not know can be an expression of risk (34). This is in contrast to existing instruments where Do not know is disregarded. Unknown patients will necessarily have many Do not know scores, and our findings indicate that the V-RISK-Y could detect risk in these patients. In everyday clinical practice, this means that Do not know scores should also be weighted in the final assessment of low, moderate or high risk. A more thorough examination of missing scores and Do not know scores and the clinical guidelines for the use of the V-RISK-Y will depend on results of larger studies preferably with a multicenter design, and where centers without prior knowledge of the V-RISK-10 are also included.

Girls constituted about 80% of the study sample. The target population comprised all admissions during a year and can be considered representative of the group of young people who are admitted. However, the excluded sample was not random, with a possible preponderance of older boys with shorter hospital stays, which reduces generalization. There was a connection between age and gender and “violent episodes” and there was a certain (but not significant) degree of confounding due to age and gender, which shows the importance of controlling for age and gender in the analysis.

The results showed a slight increase in predictive accuracy for the final assessment of Low—Moderate—High risk compared with the V-RISK-Y score alone (Table 1). Although the results are not significant, they nevertheless indicate that the final individual assessment might increase accuracy, as intended with SPJ instruments (7, 21).

Only three single items were significant in single item analyses controlled for age and sex: Items (1) Violence, (2) Threats of violence, and (7) Suspicion. Item (12) User’s (youth’s/parent’s) own assessment of risk was close to significance. The statistical significance of the other single items was weak, but relatively high ORs for many items could be of clinical interest and might provide a basis for further testing of the screener with a higher number of participants.

The odds ratio of Item 11 Trauma was below 1, which means that this item contributes negatively (albeit non-significantly) to the predictive value of the total score. This may seem somewhat surprising, but youths admitted to an acute ward constitute a highly selected sample, and many of these patients might be hospitalized due to self-harm, and not violence. A lower proportion (28%) of the scores of the item 11. Trauma were “No,” giving a high incidence of positive scores which might indicate that this item is not so well suited to identifying those at risk in an emergency setting. In outpatient and community settings, such as schools’ health and social services and municipal services where symptom pressure and selection are less pronounced, the trauma item might be more relevant for violence, in line with findings from Maas et al. (39).

The other new item, Youth’s and parent’s own assessment or perception of risk, was close to significance, in line with earlier research (23–25). As far as we know, the user’s own risk perception is not present in any existing risk instruments, and the V-RISK-Y seems to be the first risk assessment tool to implement a user (patient) item.

Despite not significant findings, from a clinical point of view it may seem surprising that only one out of four High risk youth had an episode of violence, while eight out of 18 Moderate risk youth had an episode of violence. In contrast, only two out of 44 Low risk youth were documented as engaging in violence.

However, an assessment of High risk could have led to increased awareness and precaution for the patient and closer monitoring by staff, which might have prevented violent episodes.

Further, more restrictive means were imposed on the High-risk patients (75%), compared to the Moderate risk group (44%).

The anticipated time to fill in the screener was only 6–7 min, and all staff who participated in the evaluation recommended the V-RISK-Y for use. Staff also concluded that the usability of the V-RISK-Y was equal or better compared to the V-RISK-10 that had been used previously in the department.

There were feasibility issues concerning the use of the V-RISK-Y regarding scoring the relevance category items. During data collection itself, several staff expressed uncertainty about relevance scores and particular difficulty in assessing relevance. Information to assess the presence scores was easier to obtain from the interview, observations at admission, medical records, and other sources available at admission. Assessing relevance scores would require more in-depth, detailed information about the patients and take too much time in the screening context, and the feasibility of the screener would run the risk of missing scores.

Uncertainty with the meaning of the relevance score for predictive validity has been shown for the HCR-20 (40, 41).

Assessing relevance might also have affected the low participation rate (34%) and the results of the inter-rater reliability (IRR) exercise. The average result for all items was fair, but for some items, poor. Characteristics of the short case stories, such as the fact that all cases had relatively high sum-scores on the V-RISK-Y and only three of the 90 forms concluded with low risk of violence, could have had an impact on results. The case stories may be better suited for discussions of the scores than for an examination of IRR.

### 4.1. Limitations

Limitations of our study were the pilot study design giving a small sample size and involving only one acute ward, increasing the possibility for Type II errors and random errors. There were no neutral research staff, and the treatment staff scored and recorded both baseline and outcome variables. However, closeness to patients might result in better monitoring and more information. Furthermore, as with all investigations of adverse events as outcome, one can for obvious ethical reasons not only observe what happens, but also both staff and parents/next of kin would try to avoid or prevent the outcome (violence). Hence, an accurate risk assessment may then turn out to be a wrong risk prediction, because of the efficient risk prevention that was implemented. The strengths of this study were the prospective design and the naturalistic observational setting that included all admitted patients.

### 4.2. Conclusion

We must interpret the findings within the pilot study design. The V-RISK-Y showed promising results as a screening tool for violence risk in an emergency psychiatric ward for adolescents. Still, because of the small sample size and only one acute ward, our findings cannot be generalized. Further, the results showed uncertainty with the relevance scores and that scoring relevance might not be suitable for a screening tool. Nevertheless, results were promising for presence scores, with large effect sizes for predictive validity. Staff recommended the screener. The study shows that there is a basis for further testing of a revised version of the V-RISK-Y in large-scale research, preferably with a multicenter design.

## Data availability statement

The original contributions presented in the study are included in the article/supplementary material, further inquiries can be directed to the corresponding author.

## Ethics statement

The studies involving human participants were reviewed and approved by the data protection officer at Oslo University Hospital. Written informed consent for participation was not provided by the participants’ legal guardians/next of kin because: the present study was approved without the patients’ or parents’/next to kins’ consent by the data protection officer at Oslo University Hospital (ID 20/01146). We also sought to proceed with written consent due to concerns regarding, the potential generalizability of the project. For various reasons, there might be some youths or parents who will not consent to participation, and, in some cases, informed consent could be difficult to obtain. Some people who have symptoms such as pronounced ambivalence, obsessive–compulsive disorder, or loss of reality may find it difficult to give consent because of the influence of their symptoms and functioning. They may spend a lot of time on the issue of consent, be insecure and suspicious about what consent will mean for them or be unable to form an opinion about the implications of consent. Some may be admitted against their will and become negative regarding the ward and unwilling to consent based on this. How many would not consent is difficult to predict, but the possibility that non-consenters have certain common characteristics (skewed selection, not random) will reduce generalizability. At admission or soon after, both patients and parents got written and verbal information about the study. The patients or parents had no further contact with the study—no interviews and no forms to fill in.

## Author contributions

JR: project protocol, supervision, data gathering, data analyses and interpretation, and preparing the manuscript. CG and TL: implementation, supervision, and preparing the manuscript. ØL: data analyses and interpretation and preparing the manuscript. SB: project protocol, data analyses and interpretation, and preparing the manuscript. All authors contributed to the article and approved the submitted version.

## Conflict of interest

The authors declare that the research was conducted in the absence of any commercial or financial relationships that could be construed as a potential conflict of interest.

## Publisher’s note

All claims expressed in this article are solely those of the authors and do not necessarily represent those of their affiliated organizations, or those of the publisher, the editors and the reviewers. Any product that may be evaluated in this article, or claim that may be made by its manufacturer, is not guaranteed or endorsed by the publisher.
